# The Minimum Data Set and Quality Indicators for National Healthcare-Associated Infection Surveillance in Mainland China: Towards Precision Management

**DOI:** 10.1155/2019/2936264

**Published:** 2019-07-07

**Authors:** Hongwu Yao, Jijiang Suo, Yubin Xing, Mingmei Du, Yanling Bai, Bowei Liu, Lu Li, Rui Huo, Jian Lin, Chunping Chen, Qiang Fu, Yunxi Liu

**Affiliations:** ^1^Department of Infection Management and Disease Control, Chinese PLA General Hospital, Beijing, China; ^2^HangZhou XingLin Information Technology CO., LTD, Hangzhou, China; ^3^National Institute of Hospital Administration, Beijing, China

## Abstract

The magnitude and scope of the healthcare-associated infections (HCAIs) burden are underestimated worldwide, and have raised public concerns for their adverse effect on patient safety. In China, HCAIs still present an unneglected challenge and economic burden in recent decades. With the purpose of reducing the HCAI prevalence and enhancing precision management, China's National Nosocomial Infection Management and Quality Control Center (NNIMQCC) had developed a Minimum Data Set (MDS) and corresponding Quality Indicators (QIs) for establishing national HCAI surveillance system, the data elements of which were repeatedly discussed, investigated, and confirmed by consensus of the expert team. The total number of data elements in MDS and QIs were 70 and 64, and they were both classified into seven categorical items. The NNIMQCC also had started two pilot projects to inspect the applicability, feasibility, and reliability of MDS. After years of hard work, more than 400 health facilities in 14 provinces have realized the importance of HCAI surveillance and contributed to developing an ability of exporting automatically standardized data to meet the requirement of MDS and participate in the regional surveillance system. Generally, the emergence of MDS and QIs in China indicates the beginning of the national HCAI surveillance based on information technology and computerized process data. The establishment of MDS aimed to use electronic health process data to ensure the data accuracy and comparability and to provide instructive and ongoing QIs to estimate and monitor the burden of HCAIs, and to evaluate the effects of interventions and direct health policy decision-making.

## 1. Introduction

Healthcare-associated infections (HCAIs), also known as nosocomial infections, have become an increasingly serious public health issue worldwide because of the effect on morbidity and mortality among hospitalized patients, especially in developing or resource-poor countries [1–4]. The effective prevention and control of HCAI occurrences and transmissions rely heavily on regulatory managements and coordinated interventions [5], which include comprehensive and targeted surveillance, antimicrobial stewardship program, series of infection control bundles, education and training, etc. HCAI surveillance system has been proven to be a powerful tool for estimating and monitoring the national/regional prevalence of infections and evaluating the effect of interventions, and many countries had therefore established national HCAI surveillance systems [3, 6–11], which mainly aimed to develop a Minimum Data Set (MDS) to collect standardized and uniform data about HCAIs from participating healthcare facilities to support data comparison, then to release a Quality Indicators (QIs) for use in benchmarking, public reporting, and pay-for-performance programmes. For example, the US had established a National Nosocomial Infections Surveillance (NNIS) in 1974 using a unified data collection and calculation method and aimed to compare the HCAI rates among different participating healthcare facilities and departments. And it had been proven to reduce subsequent infection rates by 32% based on an active surveillance programme with feedback to the clinicians and an infection control team [12]. Furthermore, the Danish also had established validate computer-assisted surveillance of HCAI based on selected laboratory and administrative data, which aimed at securing a follow-up on the outcome of the interventions directed at HCAIs [13]. In the last two decades, the system like MONI or TREAT has been made to develop computerized decision support systems to lower the burden of manual HAI surveillance [14]. MONI is filled with patients' administrative and raw medical data. The TREAT system allows for combination of data from different datasets and is also robust to missing data.

In mainland China, the National Institute of Hospital Administration (NIHA) established an HCAI surveillance reporting system decades ago [15, 16]. However, HCAI data was directly and manually reported by participating healthcare facilities. The collected data often lacked validity, resulting in poor data quality, unreliable comparisons, and gaps in the QIs available [17–19]. Although more and more regional health departments and healthcare facilities have started to develop electronic surveillance technology for HCAIs in recent years, the technical structures and data standards in electronic health records (EHRs) differ among healthcare facilities [18, 19]. Health data acquisition is not standardized and uniform at national level so far [20]. In other words, with the development of information technology, the current challenge in mainland China that national electronic HCAI surveillance lacks MDS and corresponding QIs is noticeable [21]. In 2013, the NIHA decided to develop a four-hierarchy system of national-provincial-municipal-institutional Nosocomial Infection Management and Quality Control Centre (NIMQCC) so as to provide an overview of the burden of HCAIs and to improve their management in mainland China [19]. The national NIMQCC (NNIMQCC) was set up, and one of its first steps was to initiate a pilot project and programme to identify MDS with corresponding QIs for a significant HCAI surveillance system at national level.

This study examines the identification and use of the MDS and QIs for national HCAI surveillance in mainland China, to help to address the national HCAI surveillance challenge and emphasize the importance of MDS and QIs in effective data utilization and quality management.

## 2. Methods

### 2.1. The Identification of MDS and QIs

Drawing lessons and experiences from both own previous surveillance and other countries' [14, 21–23], the MDS and QIs for national HCAI surveillance should be designed as a practical and efficient tool to focus on the data standard and quality at all stages of reporting process as well as the conclusion interpretations in the feedback reports. The process for developing the MDS and QIs was designed to ensure quality data covering all stages of the surveillance process to allow useful conclusions to be drawn. It therefore focused on directly collecting health process data from electronic health records (EHRs) including hospital information system (HIS), laboratory information system (LIS), electronic medical record (EMR), and radiology information system (RIS). Obviously, the process data was of continuous and traceable characteristics without any artificial errors or optional modifications. For example, we preferred to extract the dates recorded for hospitalization, transfer, and discharge from the EHRs to automatically calculate and acquire the length of hospitalization rather than the result calculated and reported by the HCAI staffs. Then crude data element and Quality Indicator lists were assessed and proposed by both HCAI experts and information technology staffs to ensure that they complied with this requirement. The crude lists were then sent to a multi-institutional and multidisciplinary expert team including hospital managers, HCAI directors, clinicians, epidemiologists, sterilizing scientists, nurses, and laboratory professors for further improvement and optimization, respectively. Meanwhile, filed trips and inquiries were initiated to investigate both informatization degrees and management challenges in different healthcare facilities. The experts' comments and investigation results were collected and summarized to assess the value and feasibility of each data item and element of MDS and QIs.

### 2.2. The Development of MDS and QIs

After repeated discussion, investigation, and validation, the final set of items and elements in MDS and QIs were agreed by the expert team. Then the NNIMQCC started two pilot projects for HCAI surveillance to examine the applicability, feasibility, and reliability of MDS and QIs, and the impact of surveillance on the HCAI management and quality control. The pilot projects performed well, and the NNIMQCC carried out a major promotion exercise in 2015 to encourage the establishment of an HCAI surveillance system in more provinces. All healthcare facilities were encouraged to improve their EHRs to ensure that they could provide all the necessary data of HCAI based on MDS. A survey checklist tool with free technical support was created to help participating healthcare facilities to find and solve technical problems with automated extraction and generation of the required data package from EHRs.

## 3. Results

The data elements of MDS and QIs were both classified into seven categorical items, and the total numbers of the ultimate data elements in MDS and QIs were 70 and 64, respectively. To provide references and explanation for MDS and QIs nationwide, a dedicated book of “guidelines for the implementation of basic data sets and quality control indicators for healthcare-associated infection surveillance” was issued in 2016.  Table 1 showed the items in the MDS, covering: basic (12 elements), birth (2 elements), diagnosis (10 elements), treatment (20 elements), microbiology (14 elements), vital signs (4 elements), and HCAI reporting (8 elements). Each data element in the MDS was described by name, numerical code, definition, category, use, correlation indicators, collection, format, permissible values, data source, extraction and description, range of extraction, and scope of exclusion of data elements.

The QIs included various indicators/rates relating to HCAIs, across infection (6 indicators), surgical site (3 indicators), device-related (6 indicators), neonatal care (6 indicators), bacterial resistance (10 indicators), antibiotic use (20 indicators), and on-site inspection (13 indicators). Apart from the indicators about on-site inspection, all could be directly obtained by calculation from the data elements in the MDS. The information about each indicator included its name, numerical code, definition, significance, source, formula, calculation, numerator, denominator, data elements from the MDS, collection, and analysis suggestions.

Two pilot projects were responsible for collecting qualified data packages on HCAIs from healthcare facilities to evaluate HCAI prevalence and provide feedbacks based on MDS and QIs. One pilot project was conducted by Shandong Provincial Hospital at the provincial level and the other was conducted by the Chinese PLA General Hospital at the trans-provincial level. Based on MDS, the data package was based on standard XML format. Data packages from participating healthcare facilities were required to upload every month. The data verification module was set before data submission to make sure that data packages were standardized and qualified. Data was analyzed by the full-time staffs and the feedbacks were mainly consisted of monthly reports, quarterly reports, and annual reports, which were about the HCAI QIs at institutional and regional level. Clearly, the pilot projects provided valuable information about the performance of MDS and QIs and also promoted the national HCAI surveillance system.

**Figure 1:**
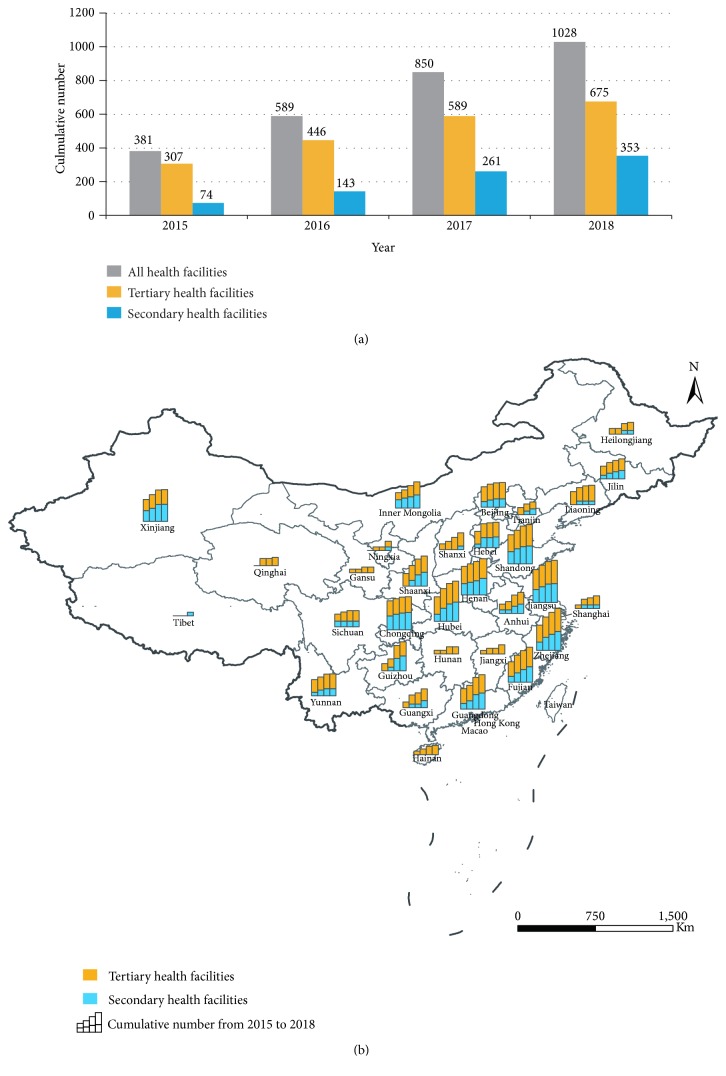
The estimated cumulative number of healthcare facilities with the ability to export the qualified data package from 2015 to 2018 at national (a) and provincial (b) level. The data in 2018 was up to September 2018. The digital province-level map of China was obtained from the Data Sharing Infrastructure of Earth System Science (http://www.geodata.cn) to produce a thematic map of the cumulative number of healthcare facilities in different provinces using ArcGIS 9.2 software (ESRI Inc., Redlands, CA, USA). The significant differences in cumulative numbers across provinces meant that the cumulative number in (b) was adjusted from primary data using a formula: y = log_2_ (x+1).

Following encouragement from NNIMQCC and promotion of the pilot projects, more and more healthcare facilities started to prepare for automated extraction of the qualified data package so as to participate in regional and national HCAI surveillance systems. This will allow them to compare their own QIs over time (intrahealthcare facility) and also benchmark against others (interhealthcare facility). These comparisons enable facilities to assess whether their interventions are successful. By September 2018, data from NNIMQCC showed there were one trans-provincial and 13 provincial HCAI surveillance systems established based on the MDS and QIs. These systems were distributed in Beijing (trans-provincial), Shandong, Hubei, Fujian, Zhejiang, Guangdong, Guizhou, Hebei, Yunnan, Shanxi, Inner Mongolia, Ningxia, Shaanxi, and Xinjiang Provinces. In total, about 485 healthcare facilities were involved, of which 200 were providing regular submissions of data packages. It is estimated that more than 1000 healthcare facilities in mainland China had realized the technology requirement for automatic extraction of the qualified data package. As shown in Figure 1, the number has increased steadily since 2015, but there are still far more tertiary healthcare facilities involved than the secondary ones. The number and level of growth varied across provinces, being fewer and slower in Tibet, Qinghai, Gansu, Ningxia, Hainan, Hunan, Jiangxi, Shanghai, and Heilongjiang Provinces.

## 4. Discussions and Conclusions

Combined use of large data resources and new technologies will solve many existing medical problems and provide better evidence for decision-making in current big data era [24]. Using big data technology to enhance health and medicine is a national priority in China [20]. Data from one health facility is more valid and more effective when it is compared with that from others [14], and up-to-date and accurate data is needed to make precision and informed decisions. The technical structures and data standards in EHRs, however, differ from health facility to health facility in most of countries. To overcome this problem, MDS has been created as a key step for establishment of surveillance system, which has become a tool used by investigators in health services research, outcomes research, and performance improvement to enhance computerized operation and identify patients with specific conditions and monitor outcomes and process measures [25–27]. For QIs, they were quantitative metric based on MDS and could provide information to improve practice, to monitor performance, to measure achievement, to determine accountability, and to define health policy decision-making [22]. In this study, we has mainly introduced MDS and QIs using for national HCAIs surveillance in mainland China, with the purpose of emphasizing the importance of MDS and QIs in effective data utilization and precise quality management.

It is well known that the HCAI surveillance system could help collect data from participating health facilities to do many data exploration and investigation. Data availability, accessibility, completeness, and validity are imperative for successful implementation of automated HCAI surveillance strategies [28]. The identification of MDS is the most basic task with regard to the data collection in the establishment of automated surveillance system [26], and the useful QIs could help reflect the reality of HCAI prevalence as well as vulnerability of HCAI management. So the availability of national HCAI surveillance with MDS and corresponding QIs is helpful and convenient for HCAI management, as well as for data sharing and comparison on institutional, regional and national level and finally on international level [14].

Although many functioning national HCAI surveillance systems have been built worldwide in recent decades, it is of much imbalance between the developed countries and developing countries [4]. What is especially true is that HCAI surveillance systems at national level are virtually nonexistent in most developing countries. Therefore, not only the health information technology, but also the HCAIs data at national level from developing countries are much scantier than those from developed countries. It is of important and urgency for countries, especially developing countries, to create MDS and QIs, and to establish a significant national HCAI surveillance system as soon as possible. In China, despite significant progress in public health and hospital care, HCAI remains a neglected challenge and economic burden [18, 19, 29]. The under-reporting HCAI prevalence and inadequate medical sources were obvious as well as imbalance healthcare facility development [19]. It was reported that missing report rate from 34.4% of investigated hospitals was greater than 60%, and nearly 50 million of the total 1.3 billion people in China required hospitalization annually because of diseases or trauma, and HCAI was associated with an annual direct economic burden of $1.5-$2.3 billion [29]. Accordingly, NNIMQCC in China focused on the identification of MDS and QIs when it started to develop a national surveillance system to lead and strengthen the precision management of HCAI and contribute to reducing its incidence and prevalence. In order to strengthen the information standardization of HCAI surveillance and solve the problem of standardization of health information in different levels and fields, taking the data elements as the key object in the research, China's Ministry of Health has promulgated a series of standards and regulations [19].

The establishment of the MDS can standardize the data collection in different healthcare facilities, improve the data quality, and ensure the good homogeneity and consistency of HCAI data arrangement and analysis. Another advantage of MDS is that the data is required to be extracted not only in a standard format but also directly from EHRs, which has greatly avoided human participation. Hence, a well-designed information technology infrastructure, data availability, accessibility, completeness, and validity are indispensable [18, 28]. The standardized HCAI data is the process data which is generated and recorded in the EHRs; it not only can better ensure the objectivity and timeliness of the collected data, but also does not need to consume too many resources for the data package exporting and uploading. So the collected data validation and QIs accuracy are guaranteed. The identification of MDS and QIs for national HCAI surveillance in China has already improved data collection, storage, release, and exchange, helping to provide consistent and comparable HCAI data and also enhance decision support for HCAI detection and resource allocation. Also providing a framework for HCAI surveillance, the structured data elements in MDS and QIs can also be of important value to implement models and simulations and other analyses.

The publication of dedicated book has greatly improved the rapid development of HCAI surveillance and supported the beginning of a national HCAI surveillance system based on information technology and computerized process data. Although the increasing trend in different provinces was various (Figure 1). This might be related to the local policies and management of the HCAI surveillance. Despite these variations, however, there is an overall increase in the use of the MDS and QIs in mainland China. In 2018, a few conferences had been organized by NNIMQCC to discuss the further development of national HCAI surveillance system; the value of HCAI surveillance system based on MDS and QIs is recognized. Next, NNIMQCC has planned to introduce related policies and regulations to continuously speed up the establishment of national HCAI surveillance system.

Generally, the HCAI burden in mainland China is increasingly severe and raised public concerns, so the NNIMQCC is responsible for establishing a well unified and definitive MDS and QIs for national HCAI surveillance system to realize the consistency and comparability of HCAI related data, as well as enhancing decision support on HCAI detection and resource allocation. The emergence of MDS and QIs indicated the beginning of the national HCAI surveillance based on information technology and computerized process data. And the publication of the dedicated book has greatly improved the rapid development of informatization in HCAI management. The identification of MDS and QIS for national HCAI surveillance in China has been proven to greatly promote the application of data collection, storage, release, and exchange, so it helps ensure the accurate and effective comparison, statistics and sharing of the information on the prevalence, prevention, and control of HCAIs in mainland China.

## Figures and Tables

**Table 1:** Summary of the Minimum Data Set and Quality Indicators for national healthcare-associated infection surveillance.

Data set	Data item	Number of data elements (*n=70*) and indicators (*n=64*)	Data element and indicator examples*∗*
MDS	Basic	12	Gender, Admission date
	Birth	2	Birth date, Birth weight
	Diagnosis	10	Admission diagnosis, Pathologic diagnosis
	Treatment	20	Operation date, ASA score
	Microbiology	14	Specimen, Antibacterial
	Vital sign	4	Temperature, Diarrhea
	HCAI reporting	8	HCAI type, Outcome
QIs	Infection	6	HCAI rate, Prevalence rate
	Surgical	3	Surgical site infection rate
	Device-related	6	Ventilator-associated Pneumonia
	Neonate	6	HCAI rate in neonate
	Bacterial resistance	10	Multidrug-resistant bacterial infection rate
	Antibiotic use	20	Utilization rate of antibiotics
	On-site-inspection	13	Omission Rate of HCAI

MDS, Minimum Data Set; QIs, Quality Indicators; HCAI, Healthcare-associated Infection; ASA, American Society of Anesthesiologists. *∗*Detailed introduction could be found in the dedicated book “Guidelines for the implementation of basic data sets and quality control indicators for Healthcare-associated infections surveillance”.

## Data Availability

This study had only used the data about the yearly number of healthcare facilities during 2015-2018 to describe the development of surveillance systems established based on the MDS and QIs (Figure 1). This data was from China's National Nosocomial Infection Management and Quality Control Center (NNIMQCC) and contained many detailed information of healthcare facilities (such as name, address, and number of beds). So the data is not publicly available now. About the data availability, readers can contact the author if they have any questions.
